# Comparison of pharmacological properties and phytochemical constituents of in vitro propagated and naturally occurring liverwort *Lunularia cruciata*

**DOI:** 10.1186/s12906-019-2534-4

**Published:** 2019-07-23

**Authors:** Sumira Mukhia, Palash Mandal, D. K. Singh, Devendra Singh

**Affiliations:** 1 Plant Physiology and Pharmacognosy Research Laboratory, Department of Botany, North Bengal University, P.O: NBU, Raja Rammohunpur, District, Darjeeling, West Bengal India; 2 Botanical Survey of India, CGO Complex, 3rd MSO Building, Salt Lake Sector I, Kolkata, West Bengal 700 064 India

**Keywords:** In vitro, Culture, Liverworts, Phytochemicals, Antioxidant, Anti-diabetic

## Abstract

**Background:**

Study of phytochemicals and pharmacological properties of bryophytes has been neglected for a long time because of the three main reasons i.e. (i) difficulty in collection in large amount for analysis; (ii) their availablility only in particular season and (iii) their restricted geographic distribution. So, the aim of this work was to propagate *Lunularia cruciata* under in vitro condition for comparing its pharmacological properties and phytocmecial constituents with naturally grown counterparts.

**Method:**

Axenic culture of *L. cruciata* was established by propagating gemmae under in vitro condition. Appropriate culture conditions, media, and the effect of hormones on growth and development were studied. The phytochemical composition was determined by GC-MS analysis and pharmacological activity was evaluated by assessing the antioxidant and anti-diabetic activities. For the antioxidant activity ABTS^+^ [2,2′-azino-bis(3-ethylbenzothiazoline-6-sulphonic acid)], DPPH^•^ (2,2-diphenyl-1-picrylhydrazyl) and metal chelating assays were done and for evaluation of the in vitro anti-diabetic activity α-glucosidase and α-amylase inhibitory activities were done.

**Result:**

Growth of *L. cruciata* was recorded in half strength MS media. Benzylaminopurine (BAP: 2 mg/L) and 1-Naphtheleneacetic acid (NAA: 0.5 mg/L) were the successful hormonal combination. GC-MS analysis revealed the existence of nine key compounds in both in vitro and naturally grown *L. cruciata*. Result of antioxidant and anti-diabetic activity showed that in vitro grown *L. cruciata* has a more or less similar antioxidant and anti-diabetic activities as naturally grown ones. This result confirms the possibility of using in vitro grown plants in place of naturally grown plants for research and clinical purposes.

## Background

Bryophytes are the second largest taxonomic group of the plant kingdom and are an integral part of biodiversity. Despite having many important ecological roles, studies on biochemical attributes of this group of plants have been neglected for a long time. In recent times numerous distinct bioactive substances have been isolated from liverworts and mosses [1–3]. Even compounds that are entirely new to plant kingdom have been obtained from this comparatively unexplored group of plants, mainly from liverworts [4]. Well expressed antibacterial, antifungal, antioxidant activity and cytotoxicity with respect to cancer cell have been demonstrated in the number of bryophytes [5–9]. Apart from this, anti-diabetic, antiplatelet, insecticidal and many other activities have been confirmed by several studies [10–12]. Traditionally some bryophyte species were applied as ethnomedicine by many tribes worldwide. In spite of being a treasure house for a large number of phytochemicals showing high biological activity, the investigations in this group are still in a nascent stage. Biochemical studies on these plants are restricted mainly due to their i) inadequate availability in nature ii) seasonal dependency and iii) habitat specificity. These impediments, however, can be successfully addressed by in vitro propagation. Although culturing plant tissues and organs under axenic conditions was first established and profitably employed in bryophytes, especially mosses [13], bryophytes did not retain for long their rightful place as a highly favored research object. Besides the problems of the establishment of axenic culture, it is often problems of material availability, the genetic variability of material, disposal of axenic organisms living on bryophytes and low level of species biology knowledge [14]. Even though researchers became successful in establishing axenic cultivation, their works are mainly focused on rare species conservation and their ex-situ reintroduction [15–18]. The works inclined towards addressing the problem of material availability for research purposes are rather rare. This study aims to compare the phytochemical composition and pharmacological properties of axenically and naturally grown liverwort *L. cruciata* to find out whether the axenically cultured bryophytes can be used as a substitute to naturally grown ones to meet up the demands of sample available for use in research purposes.

## Methods

### Plant material collection and axenic culture

Plant sample *Lunularia cruciata* was collected from Darjeeling, Eastern Himalaya, India and was identified by Dr. D.K. Singh and Dr. Devendra Singh, Botanical Survey of India. The voucher specimen was deposited in the Central National Herbarium, Kolkata [51,891/17 (CAL)]. Sporophytes of liverworts are difficult to collect in proper stage, so in this study axenic culture was initiated from gemmae. Gemmae were at first carefully taken out from gemmae cup and rinsed with distilled water. After rinsing, gammae were sterilized with 1, 2, 3 and 4% sodium hypochlorite solution for 30 s, 1, 2 and 4 min. After sterilization, gemmae were immediately rinsed with sterilized double distilled water to determine optimum concentration and exposure time of sodium hypochlorite solution. A different basal medium like such as Murashige and Skoog medium, Gamborg G5 medium and Knop’s medium were used in this study. The pH of the media was maintained at 5.8 before autoclaving and gelled using 0.8% agar. Glasswares and media were sterilized by autoclaving at 15 lb./sq. in for 15 min. Gemmae were inoculated in the media inside the laminar air flow cabinet and maintained under controlled, aseptic condition. Cultures were retained at 21 ± 2 °C under the illumination of 4000–5000 lx; alternate light and dark period of 14 and 10 h respectively. After germination, gemmae were transferred onto media with different concentrations of growth regulators:1-Naphthalene acetic acid (0.25–5 mg/L) and Benzylaminopurine (1–4 mg/L).

### Plant extracts preparation

*L. cruciata* collected from the natural and axenic condition were firstly dried and ground into a fine powder. Powdered samples were refluxed using methanol for 4 h. The extract obtained was then filtered, concentrated and used for experimental purposes.

### Gas chromatography-mass spectrometry analysis

Perkin-Elmer GC Clarus 500 system with AOC-20i autosampler and a Gas chromatograph interfaced to a Mass Spectrometer were used. The separations were done using Elite-5MS (5% diphenyl / 95% dimethyl polysiloxane) capillary tube (30 × 0.25 μm ID × 0.25 μm film thickness). Helium (99.9%) was used as the carrier gas at a constant flow rate of 1 ml for 1 min, with a split flow rate of 10.0 mL/min. The mass spectrometer was operated at 70ev ionization energy, with a scanning interval of 0.5 s, scanned from 45 to 450 m/z (mass/charge). The column temperature was maintained at 250 °C and the ion source temperature was 200 °C.

The column temperature was programmed at 110 °C for 2 min, with an increase of 10 °C/min to 200 °C, then 5 °C/min to 280 °C and finally held at 280 °C for 9 min, with the total runtime of 36 min. A sample volume of 2 μl was loaded in a mode injector. The mass detector used was TurboMass GoldTM PerkinElmer; the software used for mass spectral analysis and chromatogram was Turbo-Mass ver-5.2. MS fragmentation patterns and retention time of the compounds were compared with the NIST database. The relative percentage of compounds was quantified by comparing the average peak area to the total area.

### DPPH (2,2-diphenyl-1-picrylhydrazyl) radical scavenging activity

DPPH^•^ scavenging activity was studied by following the method of Sidduraju et al. [19]. DPPH^•^ solution was prepared by dissolving 4 mg DPPH^•^ in 100 ml methanol. The reaction was initiated by mixing 2 ml of DPPH͘ solution with 200 μl of plant extract. The reduction in the solution colour was estimated spectrophotometrically at 517 nm. DPPH͘ scavenging activity of the sample was calculated by using the following formula:

$$ \%\mathrm{I}=\left[\left({\mathrm{A}}_0-{\mathrm{A}}_1\right)/{\mathrm{A}}_0\right]\times 100,\mathrm{where}\ \left(\mathrm{I}=\mathrm{inhibition},{\mathrm{A}}_0=\mathrm{absorbance}\ \mathrm{of}\ \mathrm{blank},{\mathrm{A}}_1=\mathrm{absorbance}\ \mathrm{of}\ \mathrm{test}\ \mathrm{sample}\right). $$

### ABTS^+^ scavenging activity

ABTS͘^+^ scavenging activity of plants was estimated according to the method of Li et al. [20] with few modifications. For the preparation of 7 mM ABTS^+^ solution, 38 mg ABTS^+^ was dissolved in10 ml methanol. In a different conical flask, 6.4 mg Potassium persulphate was dissolved in 10 ml water. These two solutions were mixed together in equal proportion. The solution was then diluted eight times with water and used for the assay. In the assay 2 ml of 7 mM ABTS^+^ solution was added to 1 ml sample. The mixture was then incubated for 10 min. Absorbance was measured at 734 nm. The ABTS^+^scavenging activity was calculated by using the following formula:

$$ \%\mathrm{I}=\left[\left({\mathrm{A}}_0-{\mathrm{A}}_1\right)/{\mathrm{A}}_0\right]\times 100,\mathrm{where}\ \left(\mathrm{I}=\mathrm{inhibition},{\mathrm{A}}_0=\mathrm{absorbance}\ \mathrm{of}\ \mathrm{blank},{\mathrm{A}}_1=\mathrm{absorbance}\ \mathrm{of}\ \mathrm{test}\ \mathrm{sample}\right). $$

### Metal chelating activity

Metal chelating activity was estimated following the method of Dinis et al. [21]. At first 400 μl of the sample was mixed with 1.6 ml methanol. This step is followed by the addition of 40 μl of 2 mM FeCl_2_ and 80 μl of 5 mM Ferrozine. The mixture was then incubated for 10 min before measuring absorbance at 562 nm. Metal chelating activity was calculated by using the following formula:

$$ \%\mathrm{I}=\left[\left({\mathrm{A}}_0-{\mathrm{A}}_1\right)/{\mathrm{A}}_0\right]\times 100,\mathrm{where}\ \left(\mathrm{I}=\mathrm{inhibition},{\mathrm{A}}_0=\mathrm{absorbance}\ \mathrm{of}\ \mathrm{blank},{\mathrm{A}}_1=\mathrm{absorbance}\ \mathrm{of}\ \mathrm{test}\ \mathrm{sample}\right). $$

### α-Glucosidase inhibitory activity

The method described by Kim et al. [22] was followed with a slight modification to determine the α-glucosidase inhibitory activity of the studied plant. The reaction was initiated by mixing 2.5 ml 0.1 M phosphate buffer [pH 6.8], 0.5 ml reduced glutathione (9.2 mg glutathione dissolved in 10 ml buffer) and 0.1 ml enzyme (10 μg/ml). After 15 min of incubation, 0.5 ml of sample and 0.25 ml of ρ-NPG (30 mg ρ-NPG dissolved in 10 ml buffer) were added to the mixture. It was again incubated for 15 min and the reaction was finally stopped by adding 4 ml of 0.1 M Na_2_CO_3_. Absorbance was measured at 405 nm. α-glucosidase inhibitory activity was calculated by using the following formula:

$$ \%\mathrm{I}=\left[1-\left({\mathrm{A}}_{\mathrm{s}}-{\mathrm{A}}_{\mathrm{b}}\right)/{\mathrm{A}}_{\mathrm{c}}\right]\times 100\ \mathrm{where},{\mathrm{A}}_{\mathrm{s}}=\mathrm{absorbance}\ \mathrm{of}\ \mathrm{sample},{\mathrm{A}}_{\mathrm{b}}=\mathrm{absorbance}\ \mathrm{of}\ \mathrm{blank},{\mathrm{A}}_{\mathrm{c}}=\mathrm{absorbance}\ \mathrm{of}\ \mathrm{control} $$

### α-Amylase inhibitory activity

The α-amylase inhibitory activity was estimated following the method of Kim et al. [22]. A mixture of 0.1 ml of sample, 0.1 ml of an enzyme, and 0.3 ml of Sodium-potassium buffer (pH 6.9) was incubated for 30 min. After 30 min 0.5 ml of starch (100 mg starch in 10 ml buffer) was added to the mixture. The reaction was stopped by adding 1 ml of dinitrosalicylic acid after 5 min. Absorbance was measured at 540 nm. α-amylase inhibitory activity was calculated by using the following formula:

$$ \%\mathrm{I}=\left[{\mathrm{A}}_{540}\ \mathrm{C}-{\mathrm{A}}_{540}\ \mathrm{E}\right]/\left[{\mathrm{A}}_{540}\ \mathrm{C}\right]\times 100\ \mathrm{where},{\mathrm{A}}_{540}\ \mathrm{C}=\mathrm{absorbance}\ \mathrm{of}\ \mathrm{control},{\mathrm{A}}_{540}\ \mathrm{E}=\mathrm{absorbance}\ \mathrm{of}\ \mathrm{extract} $$

### Statistical analysis

The data were collected in triplicate. IC_50_ values and standard error of estimates were determined by using MS Excel 2007 (Microsoft, Redmond, WA, USA). Turkey’s Range Test through DSAASTAT software (version 1.002; DSAASTAT, Peruglia, Italy) was used for comparing means; *p <* 0.05 was considered to be statistically significant for differences in the mean level of components.

## Results

### Axenic culture of *L. cruciata*

Axenic culture of *L. cruciata* was initiated from gemmae as it is difficult to collect spores in the proper stage. Gemmae started germinating after 8–12 days of inoculation. Gemmae increased in size absorbing moisture and turned green in colour. Half strength Murashige and Skoog (MS) medium in alternate light (L) and dark condition (D) of 14 h L/ 10 h D was found to be most suitable, supporting the maximum number of gemmae germination. Germination of gemmae was also noticed in Knop’s micronutrient medium, but these gemmae failed to grow. Gemmae failed to germinate completely in half and full strength Gamborg B-5 medium. Continuous illumination of 4000–5000 lx and temperature 21° ± 2 °C was used. Sterilization with 1% sodium hypochlorite for 4 min or 4% sodium hypochlorite for 1 min was proved effective as gemmae remained alive and most of the microbes were killed. Exposure to a higher concentration of sterilant even for a few seconds was harmful. Similarly, treatment of the gemmae in lower concentration for a long time was also found to be lethal.

**Fig. 1:**
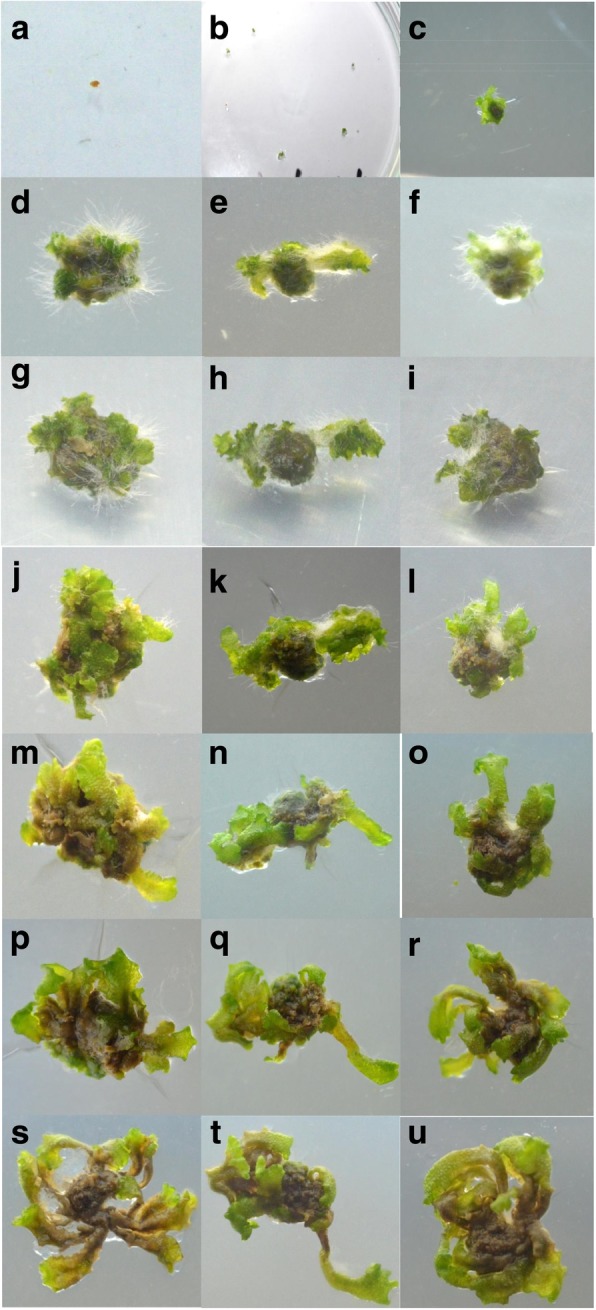
In vitro growth of *L. cruciata*. **a**. *gemmae* placed on MS media **b-c.** Germinating gemmae after 16–17 days **d-f.** Thallus and rhizoids developed from gemmae **g-i.** Well developed thalli after repeated sub culturing **j-l.** Control of excessive growth of rhizoids by alternating hormone ratio and growth of dichotomously branched thalli. **m-u** Growth and maturation of thalli (continuation)

The early sign of germination was the change in the colour of gemmae from brown to green. The highest percentage (90%) of germination was observed in half strength MS media followed by half strength Knop’s medium (20%), while no germination was noticed in full strength MS media, Knop’s medium, Gamborg B5 medium, and half strength Gamborg G5 medium. Gemmae then started to increase in size and green colour became more prominent. Young thalli started developing from gemmae after 8–12 days. After 2 weeks, thalli turned into dark blackish green coloured undifferentiated tissue called callus. At this stage, the profound effect of growth regulators like BAP and NAA was noticed. Hormone combination: 2 mg/L BAP and 0.5 mg/L NAA successfully initiated the differentiation of callus (Table 1). A higher concentration of growth regulators had shown a detrimental effect on the callus. A large number of rhizoids growing from all parts of callus were noticed in the media containing 0.5 mg/L NAA hindering further differentiation of tissues. The transfer of callus in the media containing 4 mg/L BAP and 0.2 mg/L NAA lead to the development of thallus from undifferentiated tissue with a much-reduced number of rhizoids on the dorsal side of the thallus. Under the illumination of 4000–5000 lx with alternate light and dark condition of 14 h and 10 h the thallus further continued to branch and grow (Fig. 1). Mature thallus was used to compare the pharmacological properties and phytochemical composition of in vitro grown and naturally grown *L. cruciata*.

**Table 1:** Effect of BAP/NAA on gemmae germination

	NAA (0.5 mg/L)	NAA (1 mg/L)	NAA (2 mg/L)
BAP (1 mg)	No germination	No germination	No germination
BAP (2 mg)	Germination	No germination	No germination
BAP (3 mg)	No germination	No germination	No germination

### Comparison of pharmacological activities

#### Antioxidant activity

**Fig. 2:**
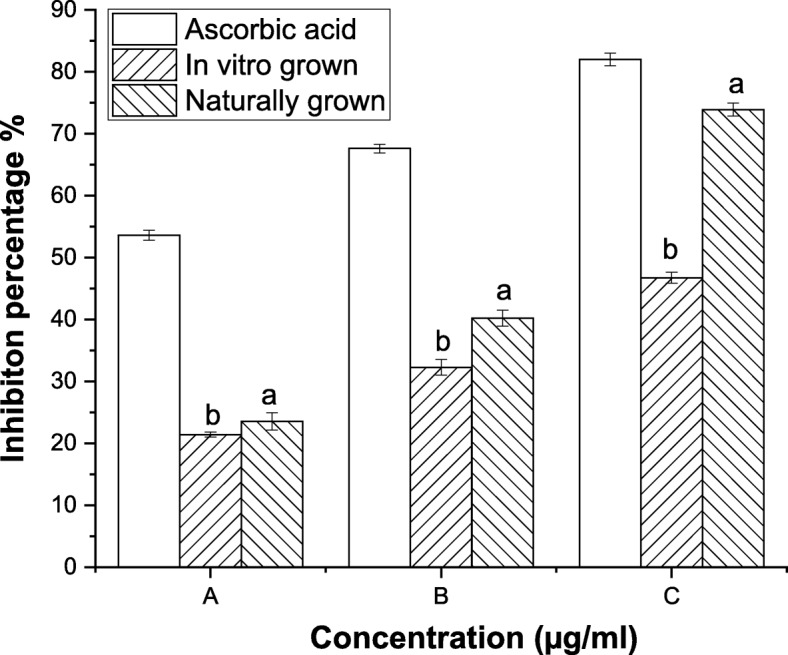
DPPH^•^ scavenging activity of in vitro and naturally grown *L. cruciata* and their comparison with standard Ascorbic acid*.* Values with different letters (a, b) are significantly (*p* < 0.05) different from each other by Turkey’s multiple range test

**Fig. 3:**
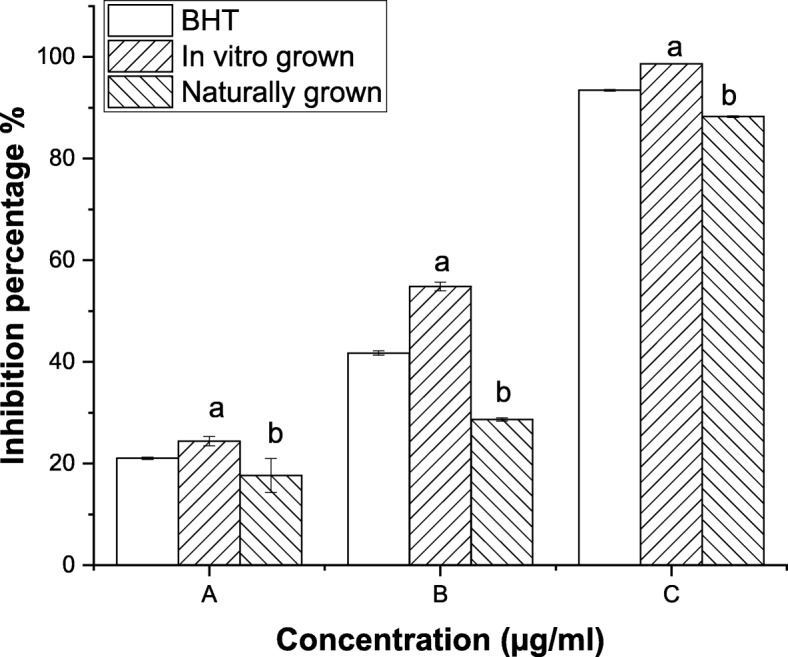
ABTS^+^ scavenging activity of in vitro and naturally grown *L. cruciata* and their comparison with standard BHT*.* Values with different letters (a, b) are significantly (*p* < 0.05) different from each other by Turkey’s multiple range test

DPPH^•^ is commonly used for investing the free radical scavenging properties of natural compounds. Naturally grown *L. cruciata* scavenged 73, 40 and 24% DPPH radicals, while, in vitro grown *L. cruciata* scavenged 48, 35 and 22% DPPH radicals at concentrations 650, 350 and 250 μg/ml (Fig. 2). ABTS^+^ scavenging assay is also widely used in the in vitro studies to assess the antioxidant property of natural compounds. In vitro grown *L. cruciata* showed higher ABTS^+^ scavenging activity than naturally grown ones. The result showed that 2 mg/ml of naturally grown *L. cruciata* extract scavenged 88% ABTS^+^ and the same concentration of in vitro grown *L. cruciata* scavenged 98% ABTS^+^ (Fig. 3).

**Fig. 4:**
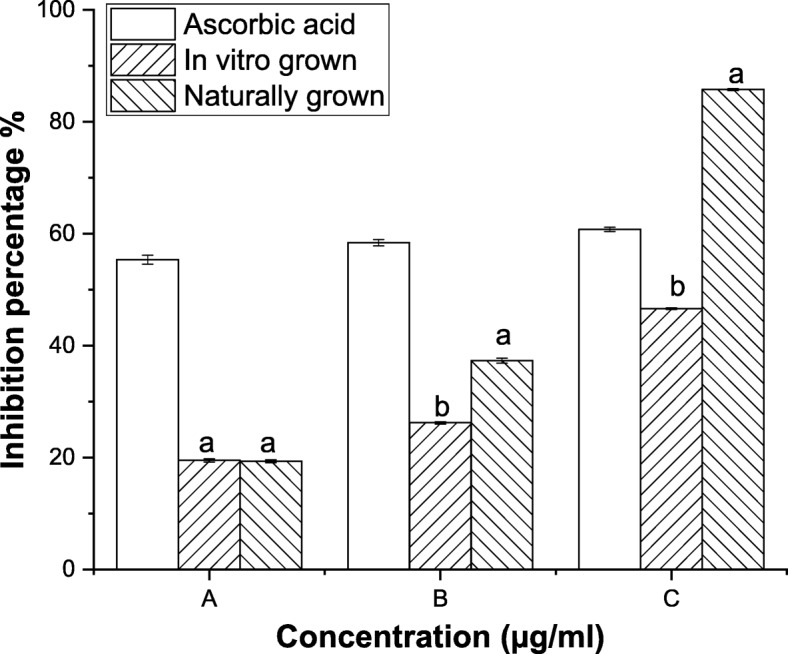
Metal chelating activity of in vitro and naturally grown *L. cruciata* with their comparison with standard Ascorbic acid*.* Values with different letters (a, b) are significantly (*p* < 0.05) different from each other by Turkey’s multiple range test

The metal chelating assay is another important antioxidant assay. Metal ions accelerate lipid peroxidation by decomposing hydrogen peroxide to alkoxyl and peroxyl radicals. Transition metal also reacts with hydrogen peroxide to form hydroxyl ion. Metal chelating activity has high perceptibility in the living system as it lowers the concentration of transition metals in the process like lipid peroxidation. Metal chelating activity of in vitro and naturally grown *L. cruciata* differed largely*.* A reduction of 85% of ferrous ion concentration was shown by naturally grown *L. cruciata* while 46% reduction in ferrous ion concentration was shown by in vitro grown *L. cruciata* when the concentration of plant sample was 4.5 mg/ml in both the cases (Fig. 4).

#### Anti-diabetic activity

**Fig. 5:**
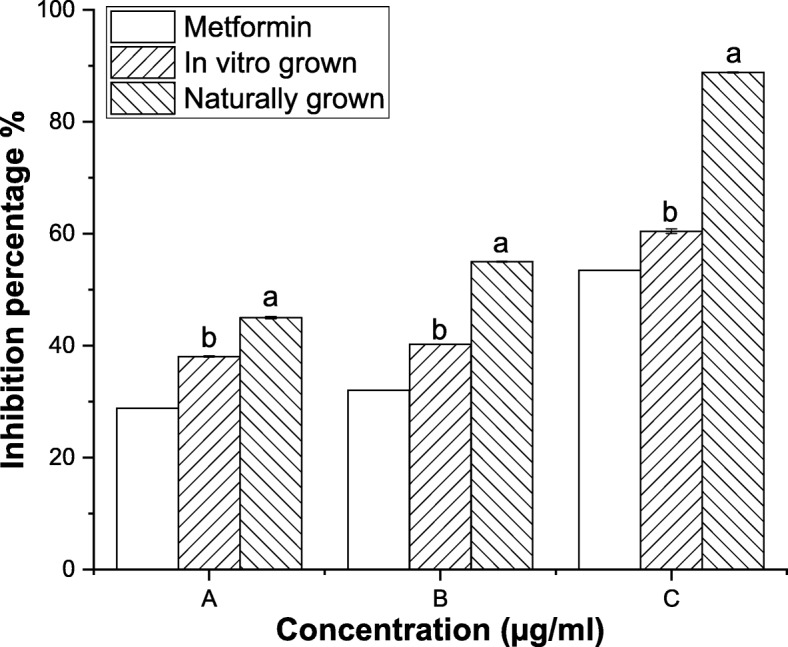
α-glucosidase inhibitory activity of in vitro and naturally grown *L. cruciata* and their comparison with standard Metformin*.* Values with different letters (a, b) are significantly (*p* < 0.05) different from each other by Turkey’s multiple range test

**Fig. 6:**
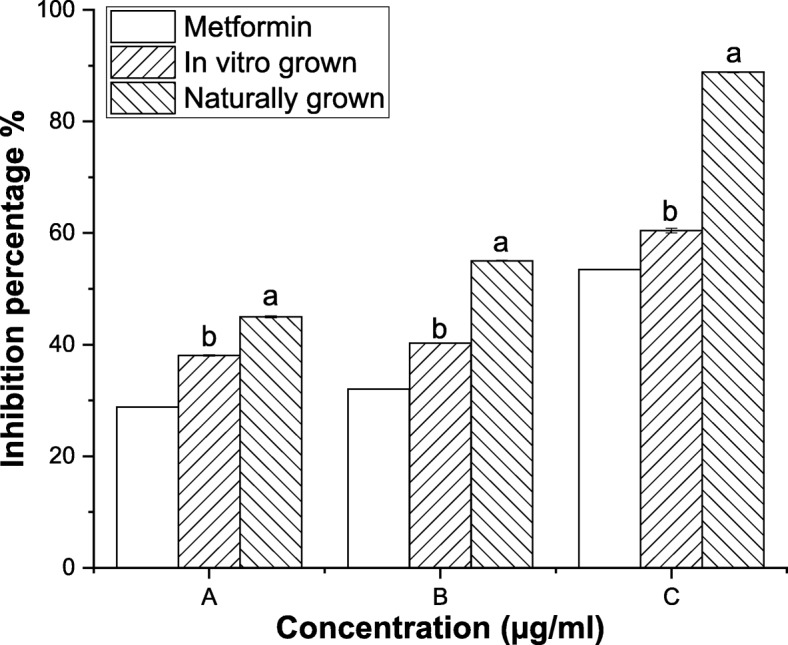
α-amylase inhibitory activity of in vitro and naturally grown *L. cruciata* and their comparison with standard Metformin*.* Values with different letters (a, b) are significantly (*p* < 0.05) different from each other by Turkey’s multiple range test

Type 2 diabetes mellitus can be managed by controlling the activity of α-glucosidase and α-amylase enzymes. *L. cruciata* obtained from both axenic and natural source showed good α-glucosidase and α-amylase inhibitory activity. In vitro grown *L. cruciata* inhibited 38, 40 and 60% α-glucosidase activity and naturally grown *L. cruciata* inhibited 44, 55 and 88% α-glucosidase activity at concentrations 0.35, 0.65 and 35 mg/ ml (Fig. 5). While, in vitro grown *L. cruciata* showed 50, 32 and 28% inhibition of α-amylase activity and naturally grown *L. cruciata* showed inhibition of 76, 70 and 68% α-amylase activity at concentrations 0.30, 0.60 and 3 mg/ml (Fig. 6).

#### Phytochemical analysis

**Fig. 7:**
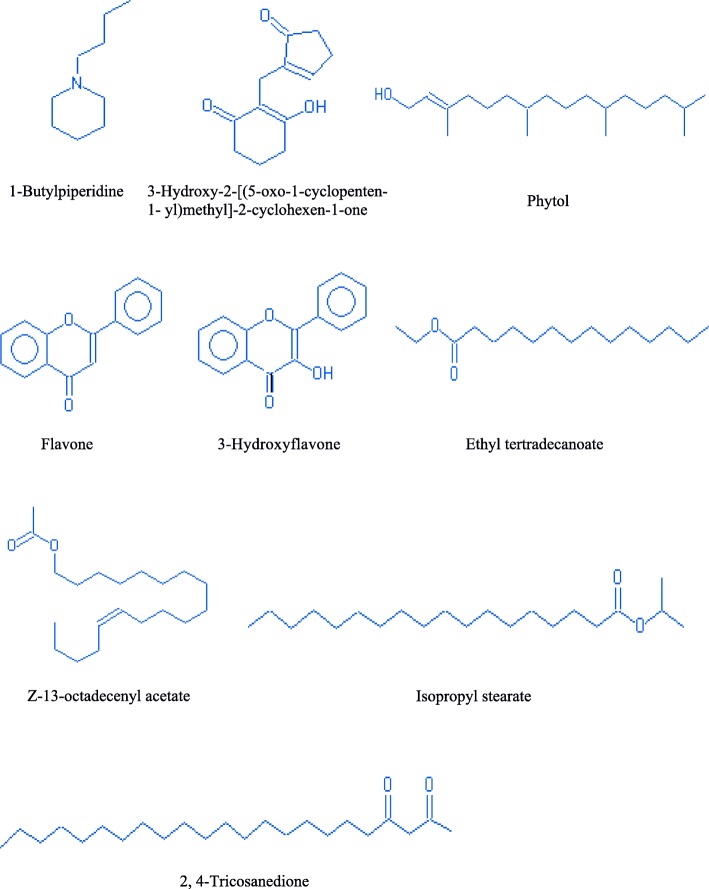
Structures of phytochemicals detected in the methanolic extract of an in vitro grown *L. cruciata*

**Fig. 8:**
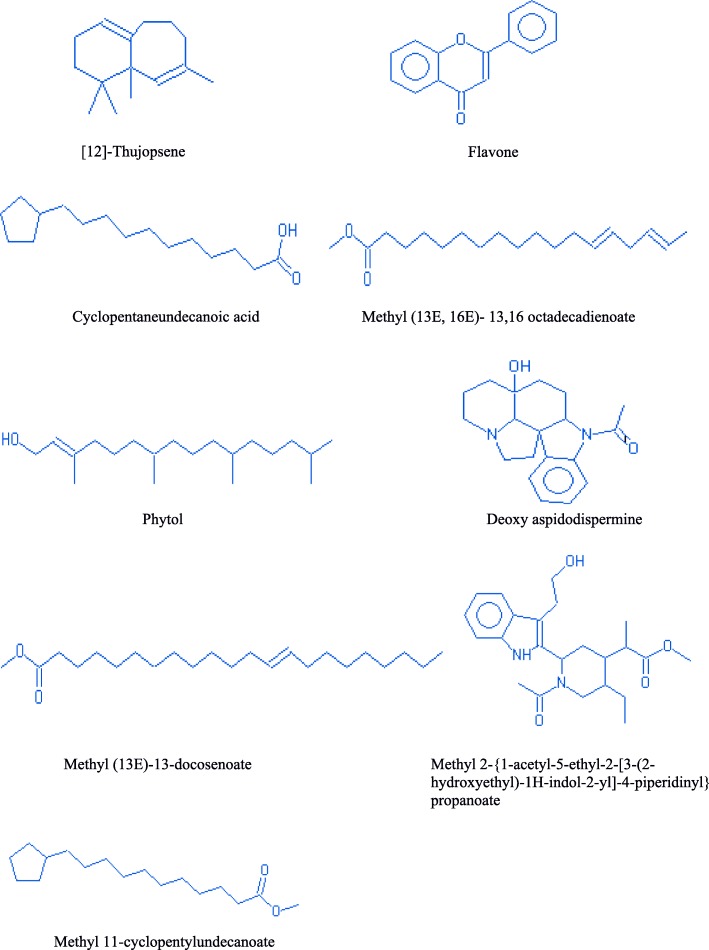
Structures of phytochemicals detected in the methanolic extract of naturally grown *L. cruciata*

**Fig. 9:**
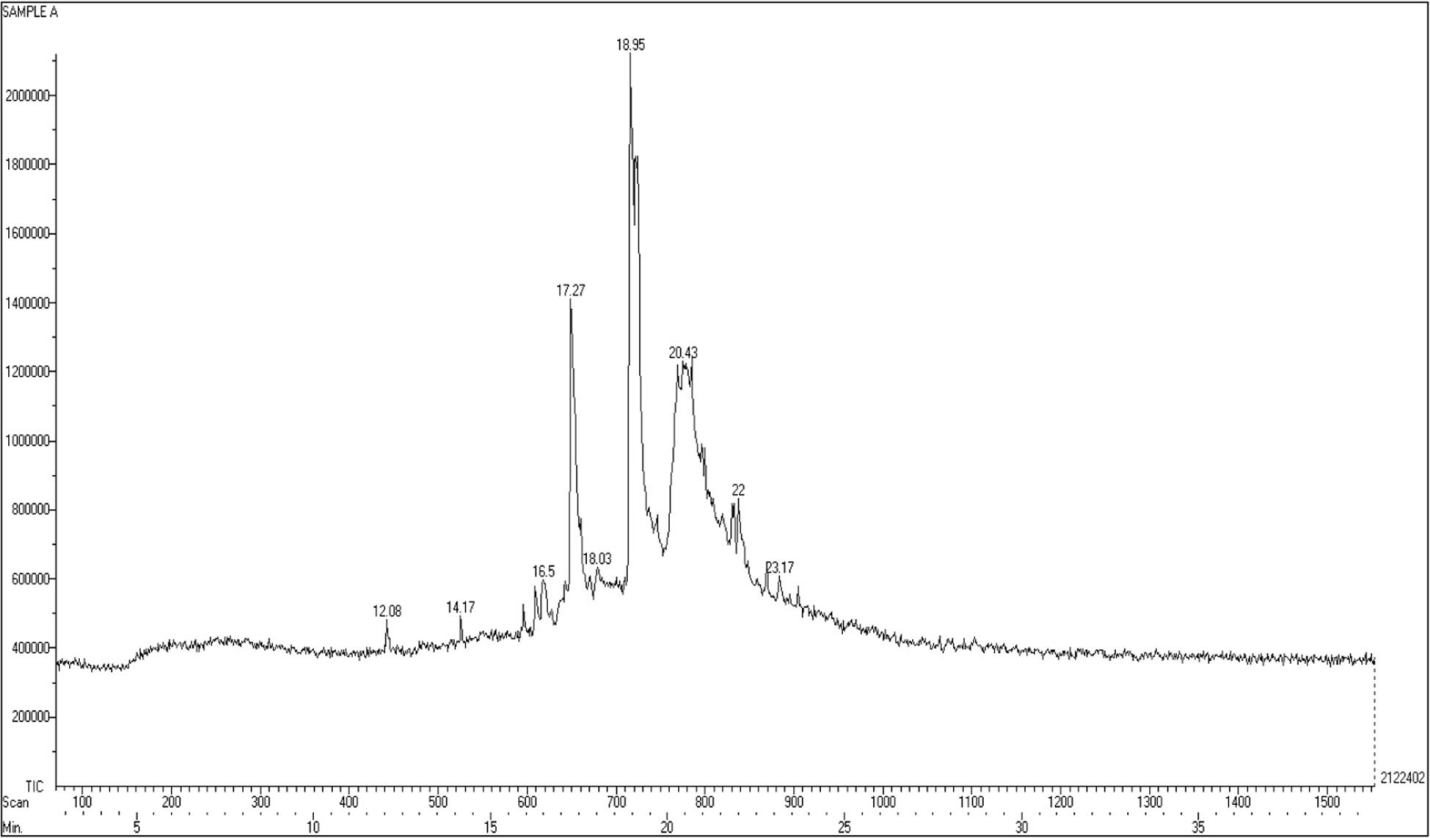
Gas chromatogram with mass-spectrometric detection of the methanolic extract of an in vitro grown *L. cruciata*

**Fig. 10:**
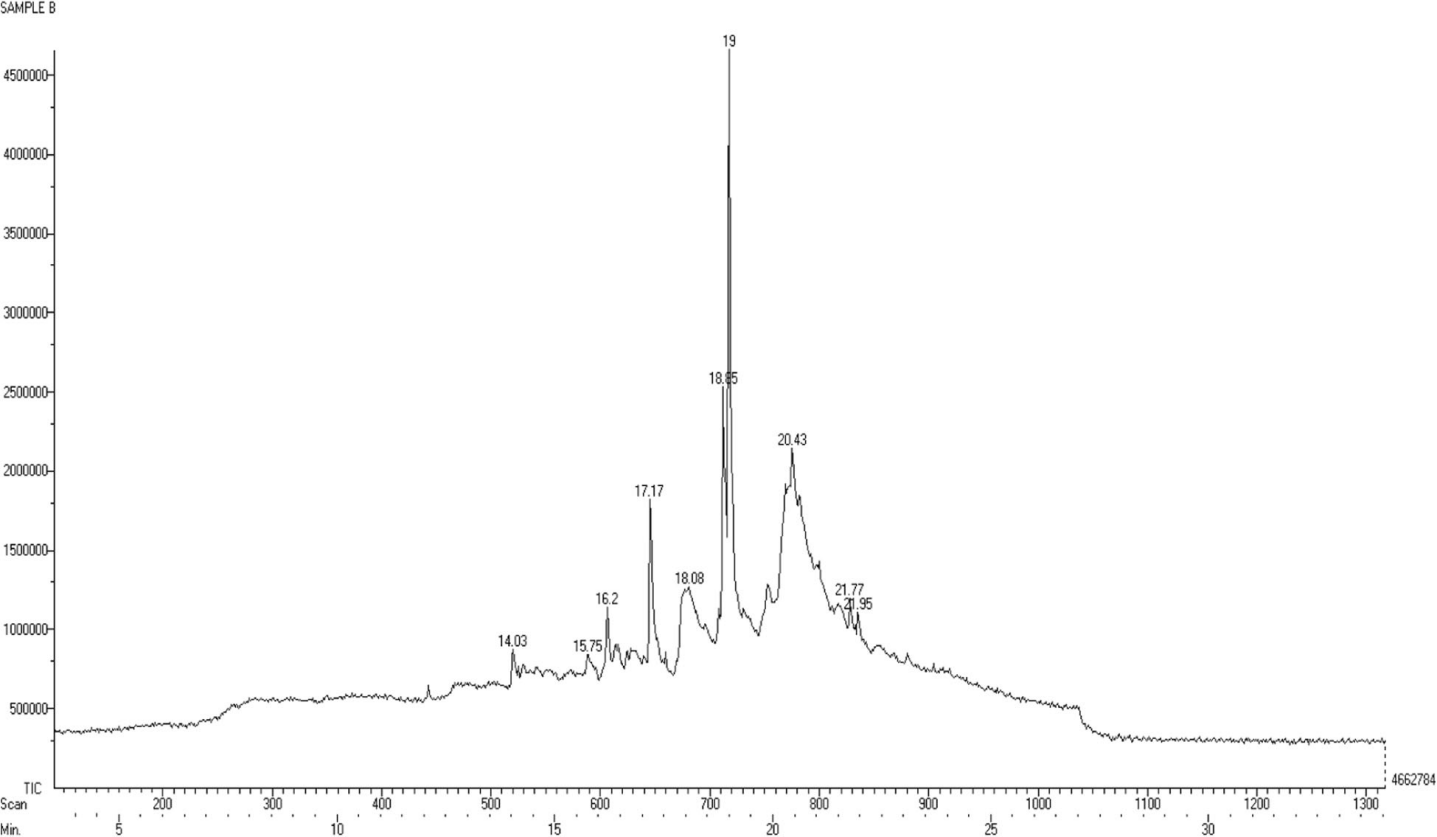
Gas chromatogrm with mass-spectrometric detection of the methanolic extract of naturally grown *L. cruciata*

The GC-MS analysis was performed to study the chemical profile of in vitro and naturally grown *L. cruciata*. Methanolic extract of in vitro and naturally grown plants showed the presence of alkaloids, flavonoids, terpenes, fatty acid, aliphatic hydrocarbon and acyclic alkanes on analyses (Figs. 7 and 8). Presence of 9 compounds each has been found in methanolic extract of naturally grown and in vitro propagated *L. cruciata* (Figs. 9 and 10). Phytochemicals present in methanolic extract of in vitro grown *L. cruciata* were 1-Butylpiperidine; 3-Hydroxy-2-[(5-oxo-1-cyclopenten-1-yl)methyl]-2-cyclohexen-1-one; flavones; 3-Hydroxyflavone, Ethyl tetradecanoate; Phytol; Z-13-octadecenyl acetate; Isopropyl stearate, and 2,4-Tricosanedione (Table 2). Phytochemicals present in methanolic extract of naturally grown plants were [12]-thujopsene; flavones; Methyl 11-cyclopentaneundecanoate; Cyclopentaneundecanoic acid; Methyl (13E,16E)- 13,16-octadecadienoate; Phytol; Deoxy aspidodispermine; Methyl (13E)-13-docosenoate, and Methyl 2-{1-acetyl-5-ethyl-2-[3-(2-hydroxyethyl)-1H-indol-2-yl]-4-piperidinyl} propanoate (Table 3).

**Table 2:** Phytochemicals present in the methanolic extract of an in vitro grown *L. cruciata* and their biological activities

Name	RT	TIC	Compound nature	Biological activities
1-Butylpiperidine	12.08	481975	alkaloid	Anti-microbial [29]; cytotoxic [30]; Antimalarial [31]
3-Hydroxy-2-[(5-oxo-1-cyclopenten-1- yl)methyl]-2-cyclohexen-1-one	14.17			No activities reported
Flavone	16.5	596325	Flavonoid	Antioxidant [36], Hepatoprotective [37], Antibacterial [38], Anti-Inflammatory [39], Anticancer [40], Antiviral Activity [41]
3-Hydroxyflavone	17.27	1412798	Flavonols	Antioxidant [36], Hepatoprotective [37], Antibacterial [38], Anti-Inflammatory [39], Anticancer [40], Antiviral Activity [41]
Ethyl tertradecanoate	18.03	632812	Fatty acid	No activities reported
Phytol	18.95	2122402	Diterpene alcohol	Antimicrobial [32], Anti-inflammatory [33], Anti cancer [34], Cardiovascular and diuretic activity [35]
Z-13-octadecenyl acetate	20.43	1229732	Aliphatic hydrocarbon	No activities reported
Isopropyl stearate	22	833849	Fatty acid ester	No activities reported
2,4-Tricosanedione	23.17	607218	acyclic alkanes	No activities reported

**Table 3:** Phytochemicals present in the methanolic extract of naturally grown *L. cruciata* and their biological activities

Name	RT	TIC	Compound nature	Biological activities
[12]-Thujopsene	14.03	873686	sesquiterpene	Antimicrobial [32], Anti-inflammatory [33], Anti cancer [34], Cardiovascular and diureticactivity [35]
Flavone	15.75	1138027	Flavonoid	Antioxidant [36], Hepatoprotective [37], Antibacterial [38], Anti-Inflammatory [39], Anticancer [40], Antiviral Activity [41]
Methyl 11-cyclopentylundecanoate	17.17	1822103	Fatty acid ester	No activities found
Cyclopentaneundecanoic acid	18.08	1274201	Fatty acid	No activities found
Methyl (13E, 16E)- 13,16 octadecadienoate	18.85	2536991	Fatty acid ester	No activities found
Phytol	19.03	2799556	Diterpene alcohol	Antimicrobial [32], Anti-inflammatory [33], Anti cancer [34], Cardiovascular and diuretic activity [35]
Deoxy aspidodispermine	20.43	2148295	alkaloid	No activities found
Methyl (13E)-13-docosenoate	21.77	1202092	Fatty acid ester	No activities found
Methyl 2-{1-acetyl-5-ethyl-2-[3-(2-hydroxyethyl)-1H-indol-2-yl]-4-piperidinyl} propanoate	21.95	1105543	Alkaloid	Anti-microbial [29]; cytotoxic [30]; Antimalarial [31]

GC-MS results have shown that in vitro and naturally grown *L. cruciata* have similar phytochemical compositions (Table 4). Phytol and flavones were present in both in vitro and naturally grown *L. cruciata*. Alkaloids were present in different forms. 1-Butylpiperidine was found in an in-vitro grown *L. cruciata* while slightly modified form, Methyl 2-{1-acetyl-5-ethyl-2-[3-(2-hydroxyethyl)-1H-indol-2-yl]-4-piperidinyl} propanoate was found in naturally grown *L. cruciata*. Fatty acid and their esters were also present in both cases. Fatty acids like Ethyl tetradecanoate and Isopropyl stearate were present in an in vitro grown *L. cruciata* while, Cyclopentaneundecanoic acid, Methyl 11-cyclopentylundecanoate, Methyl (13E)-13-docosenoate, and Methyl (13E,16E)-13,16- octadecadienoate were present in its naturally grown counterparts. However, the presence of alkanes was found only in naturally grown *L. cruciata*.

**Table 4:** Comparison of phytochemicals present in the in vitro and naturally grown *L. cruciata*

Sl. No.	Compound class		Plant growth condition				Relative abundance
		In vitro	Relative abundance	Retention time	Naturally grown	Retention time	
1	Alkaloid	1-Butylpiperidine	23%	12.08	–		
		–			Methyl 2-{1-acetyl-5-ethyl-2-[3-(2-hydroxyethyl)-1H-indol-2-yl]-4-piperidinyl} propanoate	21.95	40%
2	Terpenes	Phytol	100%	18.95	Phytol	19.03	100%,
		–			[12]-Thujopsene	14.03	31%
3	Flavonoid	Flavone	28%	16.5	Flavone	16.5	71%
		3-Hydroxyflavone	67%	16.80	–		
4	Fatty acid	Ethyl tetradecanoate	30%	18.03	–		
					Cyclopentaneundecanoic acid	18.08	46%
5	Fatty acid ester	Isopropyl stearate	39%	22			
		–			Methyl 11-cyclopentylundecanoate	17.17	65%
		–			Methyl (13E, 16E)- 13,16 octadecadienoate	18.85	91%
		–			Methyl (13E)-13-docosenoate	21.77	43%
6	Aliphatic hydrocarbon	Z-13-octadecen-1yl acetate	58%	20.43	–		
7	Alkanes	Tricosane 2,4-dione	29%	23.17	–		

## Discussion

In vitro propagation of *L. cruciata* was initiated from gemmae which started germinating after 8–12 days of inoculation. Spores of moss *Erythrodontium julaceum* and liverwort *Marchantia linearis* also required a similar time period for germination [23, 24]. Awasthi et al. [25] found a diluted nutrient medium to be the most suitable medium for germination of the spores of endangered liverwort *Cryptomitrium himalayensis*. In present work, different culture media such as Gamborg B-5, Knop, MS, and diluted MS/2 were used for optimizing micropropagation of *L. cruciata*. Among the media used, germination of gemmae was successfully initiated in half-strength Murashige and Skoog medium. The profound effect of growth regulators BAP and NAA were noticed on the growth and multiplication of thalli. Krishnan and Murugan [24] and Senarath et al. [26] used 2 mg/L BAP and 0.5 mg/L IAA for thallus and rhizoids differentiation of *Marchantia linearis*. In the present work, thallus and rhizoids differentiation from callus was initiated by supplementing basal salt media with 2 mg/L BAP and 0.5 mg/L IAA. Alteration of hormone concentration at a later stage was found to be helpful. However, excess growth of rhizoids from the thallus was noticed at a later stage that hindered the growth of thallus. Increased BAP (4 mg/L) and decreased IAA concentration (0.2 mg/L) restricted the excessive growth of rhizoids and restored normal growth and multiplication of the thallus. Alternation of concentration of growth regulators was also found to be helpful by other researchers in achieving proper growth of bryophytes in an axenic condition. Krishnan and Murugan [24] achieved root induction by transferring the differentiating leafy thalli of *Marchantia linearis* in a rooting medium containing 2 mg/L IBA. This highlighted the importance of plant hormones in controlling the growth and proliferation of plants. Young thalli then grew continuously to develop dichotomously branched fully grown thalli after 12 weeks.

**Fig. 11:**
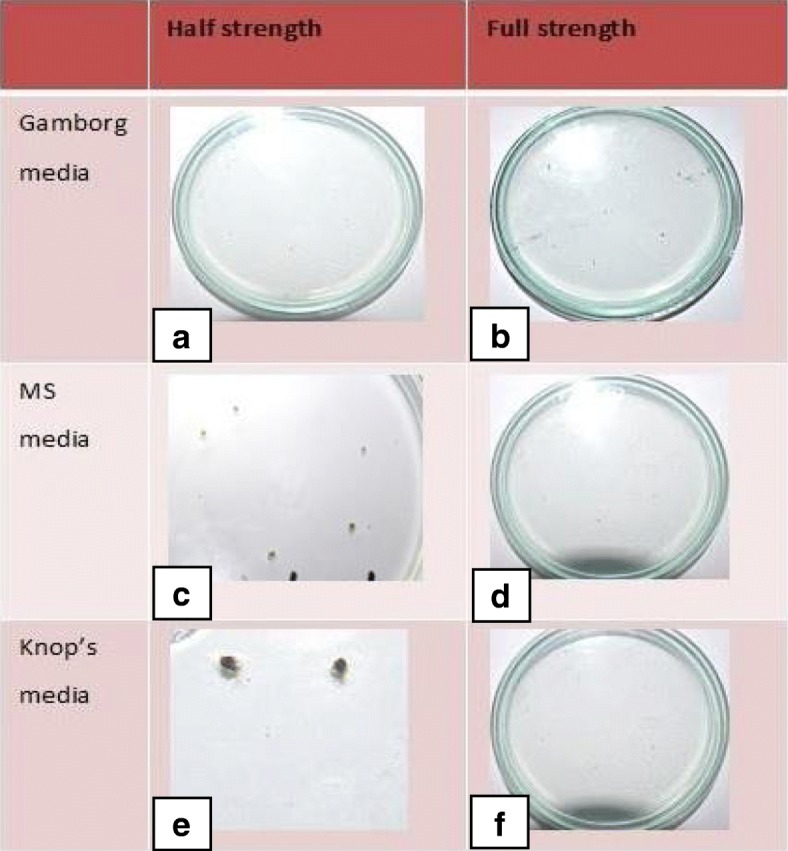
Effect of different growth media on germination of gemmae. **a**: 0% germination of gemmae; **b**: 0% germination of gemmae; **c**: 90% germination of gemmae; **d**: 0% germination of gemmae; **e** = 20% germination of gemmae; **f**: 0% germination of gemmae

In vitro propagation provides the means for rapid multiplication of plants which helps to overcome various exertions related to the isolation of phytochemicals from wild varieties due to factors like seasonal dependence, lower material availability in nature, low rates of growth and over-exploitation of wild varieties. However, there is a possibility that there occurs alteration in the phytochemical composition of naturally grown plants due to change in biochemical pathways resulting from artificial growth system [26]. Thus, comparison of phytochemicals present in in vitro and naturally grown plants is essential if the axenically grown plants are to be used as an alternative to naturally grown plants [27]. The GC-MS analysis of methanolic extract showed the presence of nine compounds each in naturally grown and axenically grown *L. cruciata*. In the present study, the presence of many similar phytochemicals was found in naturally and axenically grown *L. cruciata*. Synthesis of similar kind of phytochemicals in naturally and in vitro grown plants was also reported by Nikolova et al. [28] and Senarath et al. [26]. Alkaloids, flavonoids, terpenes, fatty acid, aliphatic hydrocarbon, and acyclic alkanes were the phytocompounds that have been detected in methanolic extract of axenically and naturally grown *L. cruciata* (Fig. 11 and Table 2). Compounds like flavone, phytol, and piperidine were common in both the plant extracts (Table 3), while, many other compounds differed slightly in structure, but belonged to same metabolite class and had similar retention time. Ethyl tetradecanoate present in an in-vitro grown and Cyclopentaneundecanoic acid present in naturally grown *L. cruciata* were fatty acids detected at similar retention time (18.03 and 18.08 respectively).

Alkaloids were detected in both the extracts. In an in vitro grown plant alkaloid was present in the form of 1-Butylpiperidine, while slightly modified ester form, Methyl 2-{1-acetyl-5-ethyl-2-[3-(2-hydroxyethyl)-1H-indol-2-yl]-4-piperidinyl} propanoate was detected in naturally grown *L. cruciata*. Studies suggest that alkaloids present in plants have anti-microbial [29], cytotoxic [30], antimalarial activities [31], etc. Thus it can be assumed that the pharmacological activities showed by axenically and naturally grown *L. cruciata* might due to the presence of these bioactive phytochemicals. Another class of compound present was terpenes. Phytol was present in both extracts while [12]- thujopsene was present only in naturally grown plants. Terpenes are reported to be responsible for many important health beneficial activities like antimicrobial [32], anti-inflammatory [33], anticancer [34], cardiovascular and diuretic activity [35]. Flavonoids like flavone were present in both naturally and in vitro grown *L. cruciata* while 3-Hydroxyflavone was present in naturally grown *L. cruciata* only. Flavonoids are important class of phytochemicals having many important activities like antioxidant [36], hepatoprotective [37], antibacterial [38], anti-inflammatory [39] anticancer [40] and antiviral activity [31, 41]. Thus it can be assumed that the pharmacological activities showed by axenically and naturally grown *L. cruciata* might due to the presence of bioactive phytochemicals like flavonoids, terpenes, and alkaloids, etc.

Fatty acid esters were also found; 2,4-tricosanedione was detected in an in vitro grown *L. cruciata* and Methyl (13E, 16E)-13,16-octadecadienoate; Methyl 11- cyclopentylundecanoate and Methyl (13E)-13-docosenoate was obtained in naturally grown *L. cruciata*.

GC-MS analysis has shown the presence of alkanes only in an in-vitro grown *L. cruciata*. In vitro grown *Fossombronia pusilla* has also produced the same terpenoid group as is produced by its natural counterparts [42]. Different fatty acid, fatty acid ester, fatty acid alcohol, and alkanes were synthesized in the in vitro grown *L. cruciata* like their natural counterparts. This suggests a possible application of in vitro cultured plants for clinical validation, bioprospection studies and commercial exploitation of novel compounds through bio farming without over-harvesting plants from their natural habitats. The presence of similar phytochemicals and biological activities in the in vitro and naturally grown plants were also reported by Vujcic et al. [43] and Senarath et al. [26].

Liverworts are moisture-loving plants with a restricted pattern of distribution growing mostly during the rainy season under natural condition [44, 45]. These drawbacks are responsible for their least use in research purposes. Also, their low availability in nature is the biggest challenge for the identification and isolation of biologically active phytochemicals insufficient amount from these plants [46]. Our finding supports the potential of in-vitro grown plants to overcome the challenges of seasonal dependence and low availability of liverworts for structural elucidation of compounds and biological assay. Potential of in-vitro grown plants to be used in place of naturally grown plants has also been reported by other researchers, like, two times increase in sesquiterpene was recorded by Otha et al. [47] in cell culture of liverwort *Calypogeia granulata*. Better production of effective phytochemical in artificial condition rather than natural habitat was also noticed by Sabovljevic et al. [48]. Different health beneficiary properties shown by herbal medicines are due to bioactive phytochemicals present in the plant [49, 50]. Presence of many phytochemicals in axenically and naturally grown plants was recorded through GC-MS analysis. In this work, antioxidative and anti-diabetic assays were performed to check whether the activity of phytochemicals of plant changes if grown in an artificial habitat. DPPH˙ is the stable organic nitrogen free lipophilic radical commonly used to investigate the free radical scavenging properties. DPPH˙ scavenging activity was observed to be superior in axenically cultured plants (Fig. 4). Increased DPPH˙ activity of in-vitro grown plants was also reported by Mohan et al. [51] in his work on *Bacopa monnieri*. Little improvement in free radical scavenging activity by in-vitro shoot extract was also reported by Manivannan et al. [27]. In a living system, free ferrous ions are the powerful pro-oxidants which cause oxidative damage and lipid peroxidation by Fenton reaction [50]. It is important to scavenge such radicals from the body of a living organism. On testing the in-vitro and naturally grown *L. cruciata* for the metal chelating property we have found that plant grown on both habitats have developed ability to scavenge ferrous ion. In this assay Fe^2+^ and ferrozine form complexes to produce hydroxyl radical, the chelating effect observed might be due to the interference of plant phytochemicals in the complex formation. Free radical scavenging property might be attributed to the presence of phenolic compounds like flavone, 4H-1-Benzopyran-4-one, 3-hydroxy-2-phenyl present in the extracts. Phenolic compound acting as radical scavengers has also been reported by workers like Wong et al. [52] and Tusevski et al. [53]. Apart from the phenolic group, other bioactive metabolites like alkaloids, terpenoids identified might have contributed to biological activities. Reduction in insulin sensitivity and postprandial hyperglycemia are the characteristics of type 2 diabetes [54]. Lowering postprandial hyperglycemia can be an important measure to control diabetes. Postprandial hyperglycemia can be controlled by inhibiting the activity of enzymes α-amylase and α-glucosidase (carbohydrate hydrolyzing enzymes) [55]. In-vitro and naturally grown plants inhibited the activity of α-amylase and α-glucosidase enzymes to an impressive level (Fig. 6). Slightly better α-glucosidase inhibitory activity was recorded in naturally grown *L. cruciata* in comparison to its in vitro grown counterparts. While, α- amylase activity was better in case of naturally grown plants.

## Conclusion

The number of researches published related to phytochemical constituents and pharmacological properties of bryophytes are considerably low. The main reason that restricts the investigation on this group is the problem of sample availability in sufficient amount to carry on analysis of bioactivity with purified compounds obtained from bryophytes. In this work, *Lunularia cruciata* was grown under in-vitro conditions as an attempt to solve the problem of geographic and seasonal restriction of sample availability. Further the phytochemical composition and pharmacological properties of naturally grown and in vitro grown *L. cruciata* was compared to record changes in pharmacological properties and phytochemical composition. Result of the study showed no significant changes in the pharmacological properties of axenically cultured and naturally grown liverworts. Phytochemical constituents of two differently grown *L. cruciata* also did not vary much. Thus the results of this study validated the use of in vitro cultured plants as a substitute for naturally grown plants to overcome the shortcomings restricting the use of liverworts for therapeutic applications.

## Data Availability

All data generated or analyzed during this study are included in the manuscript and the raw data of the analysis will be made available on request.
